# Tuberculosis Presenting as Hepatic and Splenic Microabscesses

**DOI:** 10.7759/cureus.8247

**Published:** 2020-05-23

**Authors:** Saurabh Gaba, Ashish Dua, Nayana Gaba, Monica Gupta, Suraj Agrawal

**Affiliations:** 1 General Medicine, Government Medical College and Hospital, Chandigarh, IND; 2 Radiology, Government Medical College and Hospital, Chandigarh, IND; 3 Obstetrics and Gynaecology, Postgraduate Institute of Medical Education and Research, Chandigarh, IND; 4 Internal Medicine, Postgraduate Institute of Medical Education and Research, Chandigarh, IND; 5 Internal Medicine, Government Medical College and Hospital, Chandigarh, IND

**Keywords:** tuberculosis, tb, abdominal, liver, spleen, abscess, microabscess

## Abstract

Tuberculosis (TB) is a major public health problem in developing countries. It can affect any organ of the body, and can have a multitude of clinical presentations. We present the case of a 22-year-old male who presented with fever, abdominal pain, and weight loss, and was found to have enlarged liver and spleen, both studded with multiple microabscesses. He had deranged liver functions, mild anemia, and elevated acute phase reactants. Examination of the aspirate from the liver did not reveal any organism on microscopy or culture. Based on the demographics, history of contact with a TB patient, positive Mantoux test, and clinical and radiological features, the patient was successfully managed with empirical treatment for TB.

## Introduction

Tuberculosis (TB) is a major public health problem in South Asia. The factors responsible for high incidence in developing countries are overcrowding, poverty, malnutrition, and poor sanitation [1]. India carries the highest disease burden with reported incidence and mortality of 199 and 32.72 per 100,000 population, respectively, in 2018. The prevalence of latent TB is much higher with 40% of the entire population affected [2]. The most common site of affliction is the lung, which can occur in isolation or along with other organs. The sites of extra-pulmonary disease can be lymph nodes, urogenital tract, central nervous system, abdomen, pericardium, bone marrow, eyes, skin, bones, and joints [3]. Herein, a case of abdominal TB affecting the liver and spleen in the form of multiple microabscesses is presented.

## Case presentation

A 22-year-old male, who worked at a hardware store, presented to the outpatient department with history of fever up to 102° F with night sweats, abdominal pain, anorexia, and weight loss of 4 kg over the previous month. He had progressively increasing fatigue and had to be brought in on a wheel chair. The abdominal pain was non-colicky and present diffusely over the upper abdomen. It had no association with meals, and was not exacerbated or relieved with changes in body posture. There was no history of cough, breathlessness, hemoptysis, chest pain, headache, altered bowel habits, dysuria or rash. He did not smoke and had no health problems in the past. There was no history of any substance abuse or intake of any immunosuppressive medication.

He lived in the outskirts of the city with his parents and sister in a two-room house with poor ventilation. His parents were apparently healthy, but his sister had suffered from pulmonary TB a year earlier, for which she was treated with anti-tubercular therapy (ATT) for six months. There was no significant history of travel and they owned no pets.

On examination, he appeared dehydrated and pale with blood pressure of 110/70 mm of Hg, and pulse of 90 beats per minute with regular rhythm. There was no evidence of respiratory distress or cyanosis, and the respiratory rate was 14 breaths per minute with peripheral capillary oxygen saturation of 99%. There was no jaundice, edema or palpable lymphadenopathy. The abdominal examination revealed tender hepatomegaly, so a detailed examination was deferred. The breath sounds were vesicular in character and no added sounds were present. Cardiovascular examination did not reveal any abnormality.

**Figure 1:**
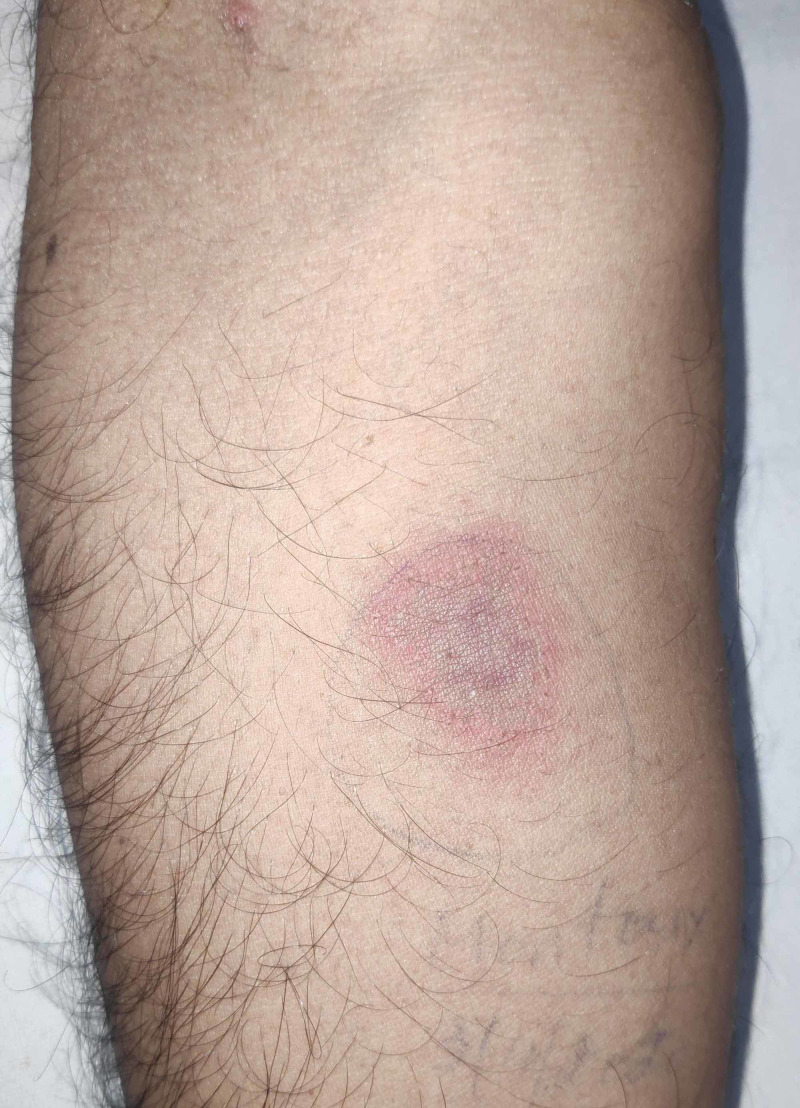
Positive Mantoux test Induration (24 mm) with a ring of erythema over the forearm, 72 hours after intradermal injection of 0.1 mL of purified protein derivative. In patients with no known risk factors for tuberculosis, induration of 15 mm or more is considered as a positive test.

The laboratory investigations revealed mild anemia, raised erythrocyte sedimentation rate (ESR) and C-reactive protein (CRP), and deranged liver functions with disproportionately elevated alkaline phosphatase (ALP) (Table 1). Blood and urine cultures were sterile. Human immunodeficiency virus (HIV), hepatitis B surface antigen (HBsAg) and anti-hepatitis C antibody (anti-HCV) were negative. Mantoux test was positive with an induration of 24 mm after 72 hours (Figure 1).

**Table 1:** Laboratory investigations

Investigation	Value	Normal range
Hemoglobin (g/dL)	10.1	13-16
Mean corpuscular volume (fL)	78	83-101
Platelets (X10^9/L)	180	150-400
Total leucocyte count (X10^9/L)	10 (80% neutrophils, 15% lymphocytes, 3% monocytes, 2% eosinophils)	4-12
Bilirubin (mg/dL)	1.3	0.2-1
Alkaline phosphatase (U/L)	1051	30-150
Gamma glutamyl transferase (U/L)	206	<50
Aspartate transaminase (U/L)	227	10-40
Alanine transaminase (U/L)	127	10-40
Total protein (gm/dL)	7.3	6-8
Albumin (gm/dL)	3.2	3.5-5.5
Globulin (gm/dL)	4.1	2-3.5
Prothrombin time (seconds)	16	14 (control)
Sodium (mmol/L)	135	135-145
Potassium (mmol/L)	4.1	3.5-5.5
Urea (mg/dL)	53	15-40
Creatinine (mg/dL)	1.3	<1.3
Glycosylated hemoglobin	5.2%	<6%
Erythrocyte sedimentation rate (ESR) (mm in 1^st^ hour)	72	<10
C-reactive protein (CRP) (mg/L)	82	<5

**Figure 2:**
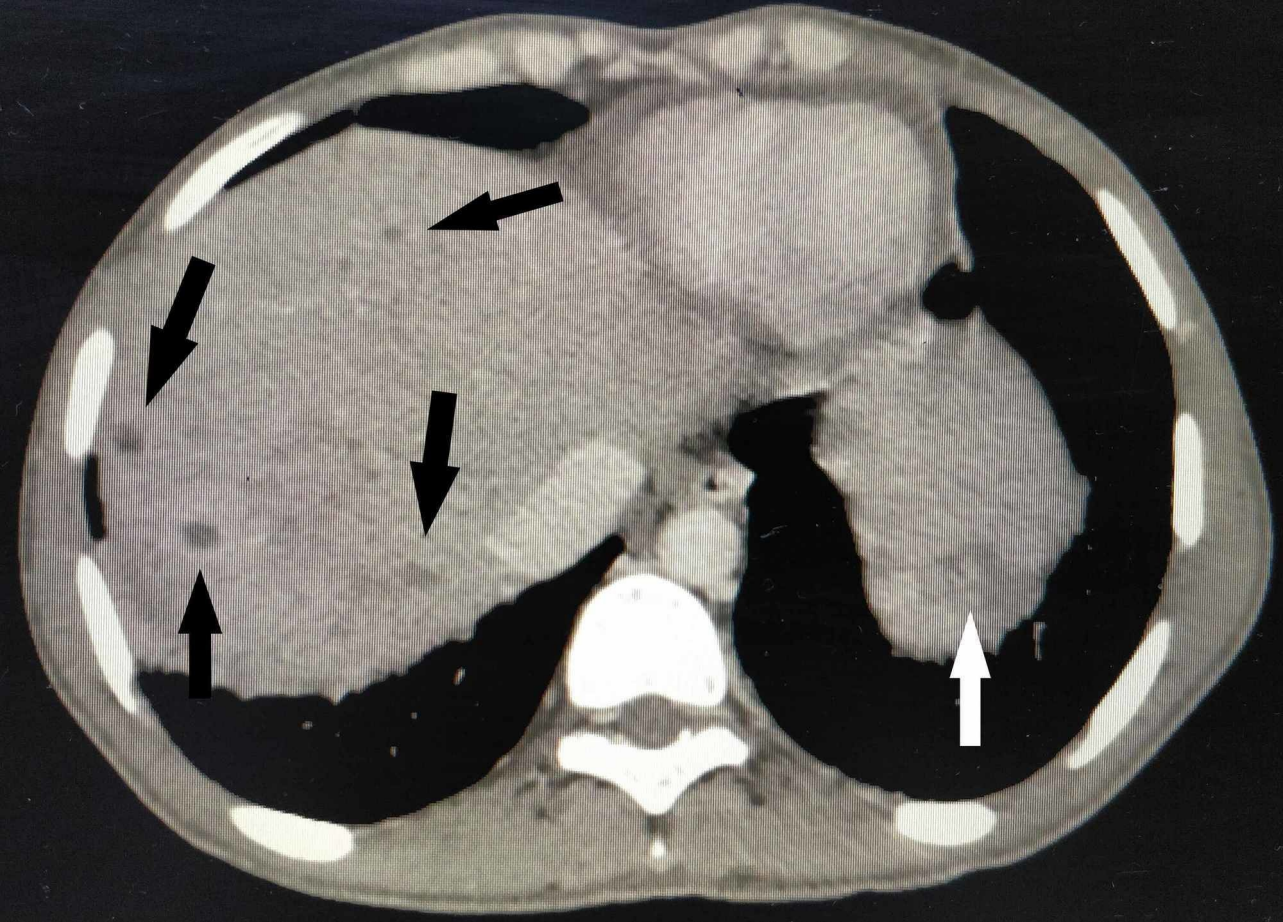
Axial section of contrast-enhanced CT scan of the abdomen Microabscesses in the liver (black arrows) and spleen (white arrow) visible as small round, hypodense lesions with minimal peripheral enhancement.

**Figure 3:**
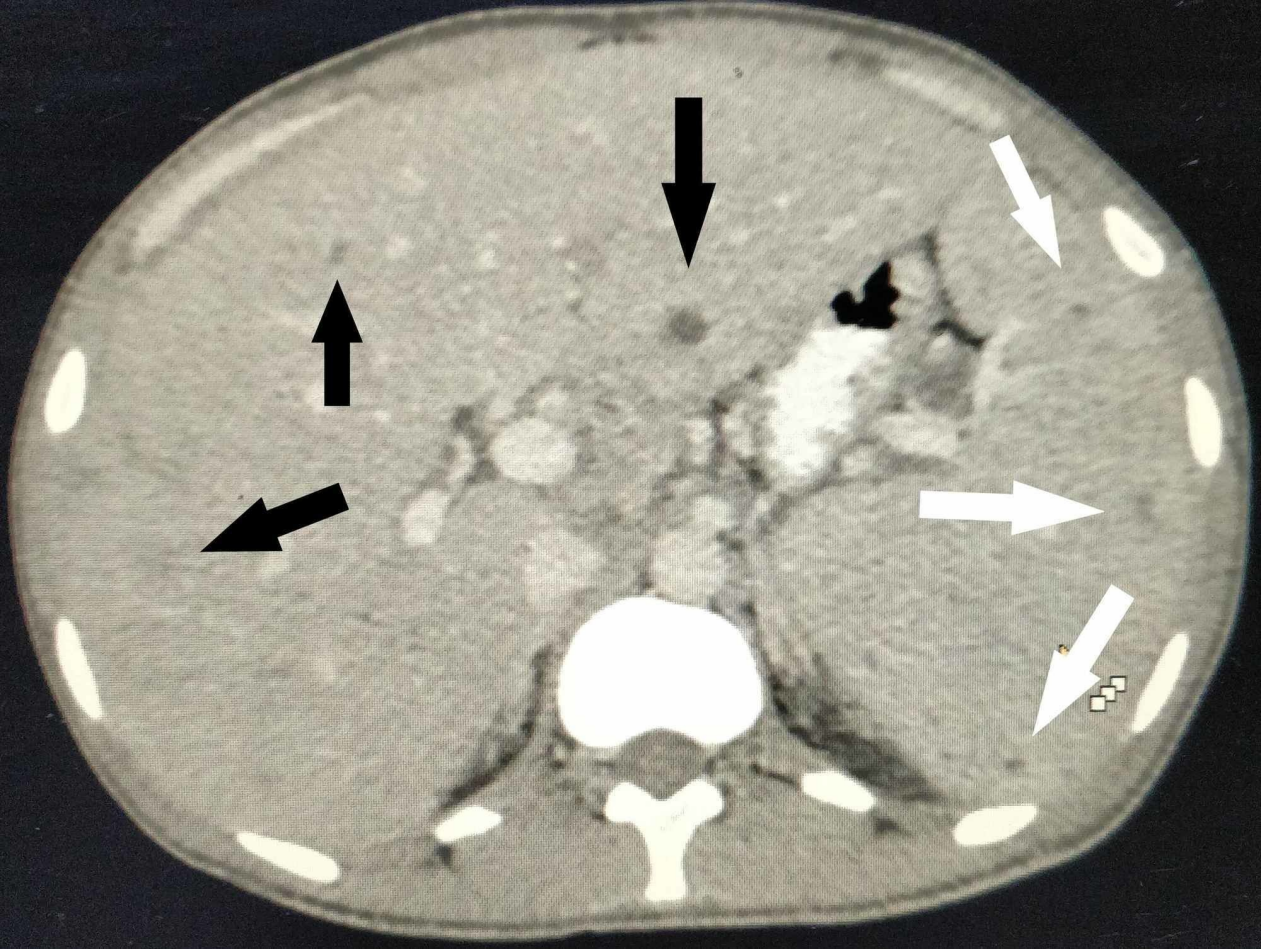
Axial section of contrast-enhanced CT scan of the abdomen Microabscesses in the liver (black arrows) and spleen (white arrows) visible as small round, hypodense lesions with minimal peripheral enhancement.

**Figure 4:**
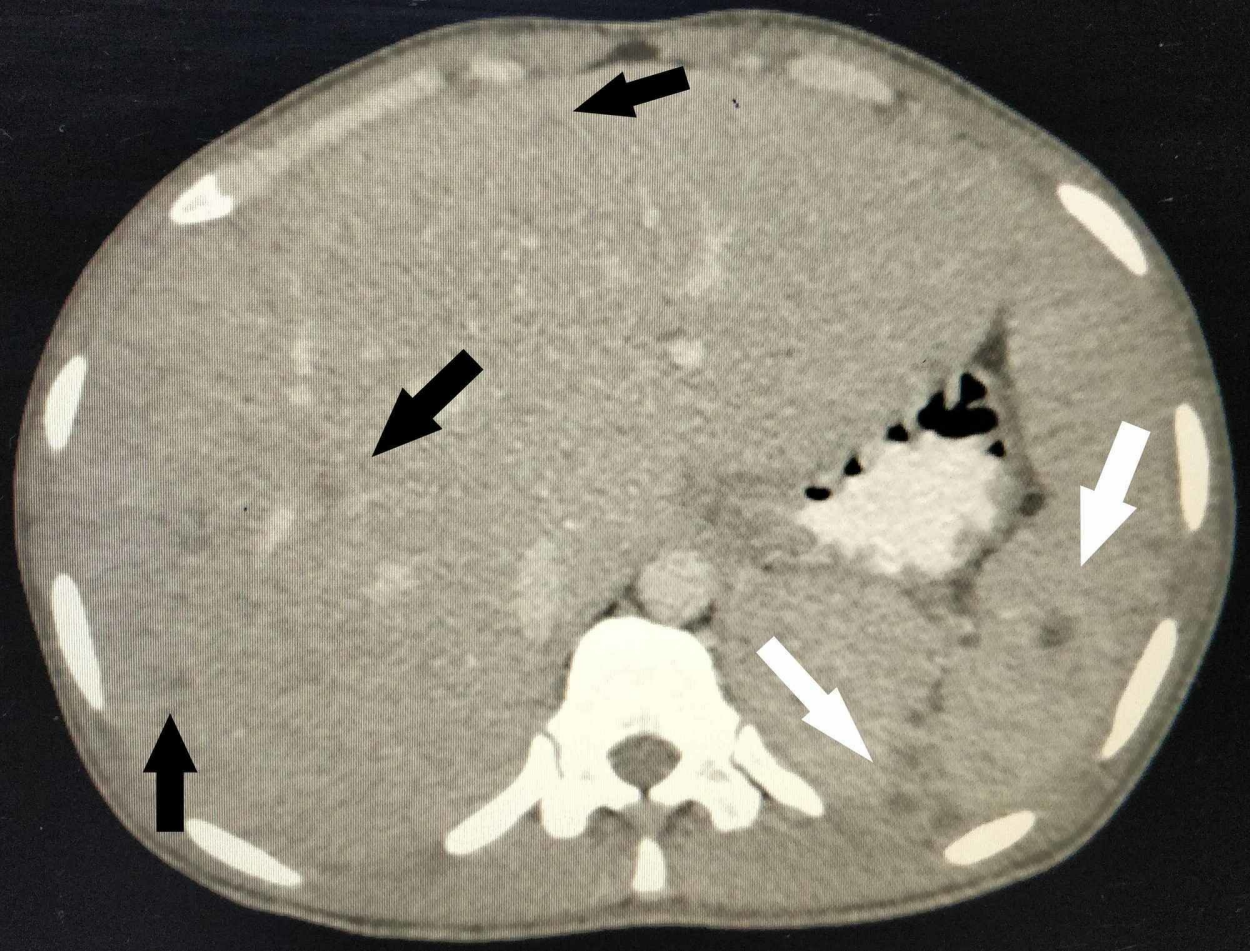
Axial section of contrast-enhanced CT scan of the abdomen Microabscesses in the liver (black arrows) and spleen (white arrow) visible as small round, hypodense lesions with minimal peripheral enhancement.

**Figure 5:**
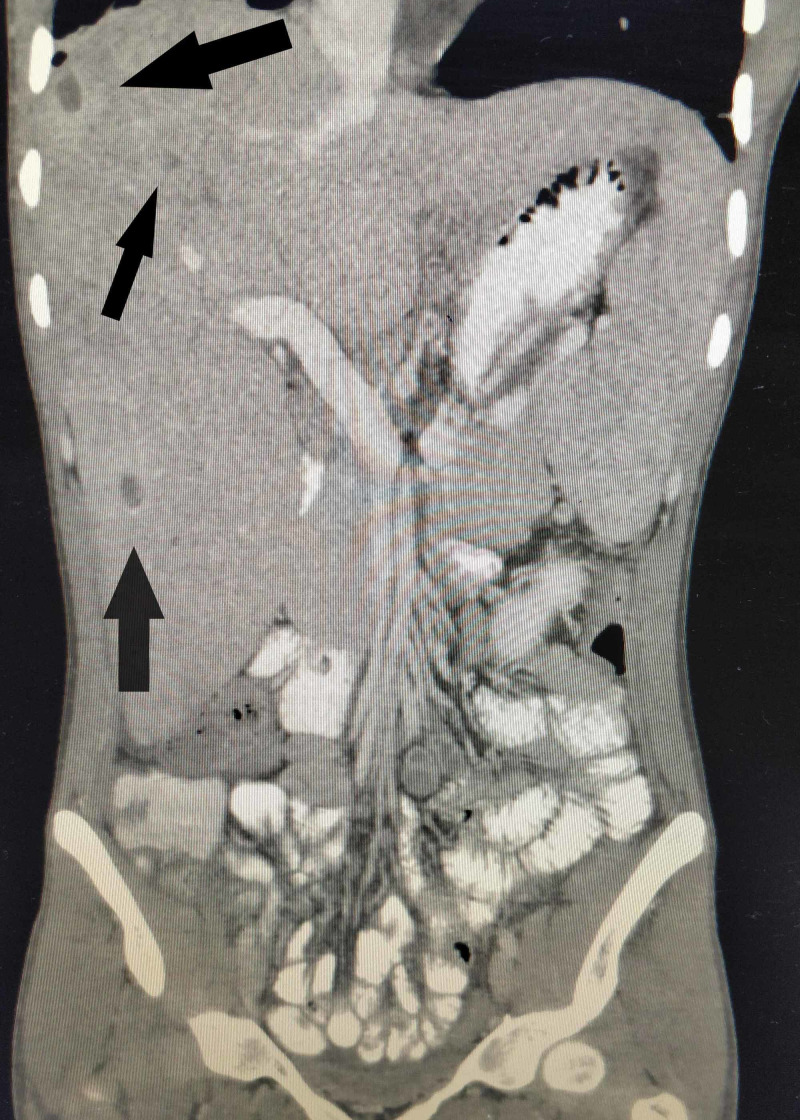
Coronal section of contrast-enhanced CT scan of the abdomen Microabscesses in the liver (black arrows) visible as small round, hypodense lesions with minimal peripheral enhancement.

A plain radiograph of the chest did not show any abnormality. Ultrasound of the abdomen revealed an enlarged liver (20 cm) and spleen (15 cm) with tiny hypoechoic foci. This was followed by a contrast-enhanced computed tomographic (CT) scan of the abdomen. The findings are illustrated in Figures 2-5. 

Ultrasound guided fine needle aspiration was performed from a hepatic lesion. It displayed necrotic hepatocytes within inflammatory infiltrate. Acid fast bacilli (AFB) staining did not reveal any organisms. Bodyweight based multi-drug regimen for TB was started, consisting of isoniazid 300 mg, rifampicin 600 mg, pyrazinamide 1500 mg and ethambutol 1200 mg a day. It was supplemented with pyridoxine 10 mg a day. The treatment was well tolerated, and was followed by defervescence within two weeks. His appetite increased and he started gaining weight. The culture of the hepatic aspirate did not grow tubercle bacilli at the end of six weeks. The liver function tests normalized within two months, and the liver and spleen size deceased on ultrasound with no visible parenchymal lesions.

## Discussion

TB can occur in virtually any abdominal structure (Table 2) [4,5]. The incidence of abdominal TB is estimated to be 11% of the total load of extra-pulmonary TB [6]. It occurs by hematogenous or contiguous spread from other organs, or by reactivation of latent TB [7]. Less commonly, ingestion of unpasteurized milk containing tubercle bacilli is implicated. Up to 25% of the patients also have pulmonary TB [8]. Certain risk factors for developing abdominal TB have been identified. These include diabetes mellitus, cirrhosis, HIV infection, peritoneal dialysis, and use of anti-tumor necrosis factor (anti-TNF) drugs [3].

**Table 2:** Radiological and clinical features of abdominal tuberculosis

Structure	Radiological features	Clinical features
Intestine	Ulcerative or hypertrophic lesions, concentric thickening of ileo-caecal region, strictures, obstruction, fistulae, lymphadenopathy, mass in right iliac fossa	Abdominal pain, fever, weight loss, diarrhea, constipation, vomiting
Peritoneum	Peritoneal thickening, omental thickening, mesenteric thickening, ascites (with fibrotic adhesions, if longstanding), lymphadenopathy	Abdominal distension, pain, fever, weight loss
Liver	Miliary nodules or solitary nodule, hepatomegaly, lymphadenopathy, biliary stricture	Fever, abdominal pain, weight loss, jaundice
Spleen (rare)	Miliary nodules, abscess, splenomegaly	Fever, abdominal pain, weight loss
Stomach (rare)	Miliary nodules, ulcers, gastric outlet obstruction	Fever, vomiting, dyspepsia, abdominal pain, weight loss
Oesophagus (rare)	Mass with ulceration or stricture formation	Fever, weight loss, dysphagia, dyspepsia
Pancreas (rare)	Mass, abscess, cystic lesion, lymphadenopathy	Fever, abdominal pain, weight loss, jaundice, malabsorption

In their review of hepatic TB patients, Hickey et al. found that the most common symptoms were fever (67%), abdominal pain (59.5%), and weight loss (57.5%) [9]. Concomitant respiratory symptoms were present in 66% of the cases. Elevation of alkaline phosphatase and gamma glutamyl transferase was prominent. 79% of the cases had miliary disease, consisting of widespread lesions, around 1 mm in diameter, that are seeded from the lungs hematogenously. The other 21% of the cases had local disease with larger lesions located near the portal triad, some showing calcification and peripheral enhancement on CT scan. These lesions can produce mass effect, leading to cholestatic jaundice. They concluded that 1% of all patients with active TB have liver involvement. Spleen is commonly involved in miliary TB, but its involvement in seclusion is exceedingly rare. Its involvement has also been reported in the form of an abscess, and as a contrast-enhancing mass that can mimic a neoplasm [10,11].

The diagnosis is established by imaging and microscopic or molecular detection of the pathogen Mycobacterium tuberculosis. Although ultrasound and contrast barium studies may suffice in some cases, a contrast-enhanced CT scan is the ideal investigation [7]. It can delineate all the lesions and the most appropriate one can be selected for tissue diagnosis. It can be obtained by radiologically guided percutaneous aspiration, endoscopy or percutaneous solid organ biopsy. Appropriate staining can reveal the AFB. Histopathology can reveal the characteristic non-caseating granulomatous inflammation, though it is not specific. Although culture can take a minimum of 2-4 weeks, it provides the opportunity for drug sensitivity testing. Nucleic acid amplification by polymerase chain reaction (PCR) can give confirmatory diagnosis within hours. Ascitic fluid is lymphocyte predominant with, in the absence of cirrhosis, low serum to ascites albumin gradient (less than 1.1 mg/dL) and adenosine deaminase (ADA) higher than 30 - 40 IU/L [12]. All the aforementioned tests carry variable sensitivity and, in the event of failure to demonstrate the tubercle bacilli, the final diagnosis can still be arrived at by careful consideration of the demographics, clinical and radiologic features, exclusion of alternate diagnoses and the response to empirical treatment, as was done in the case under consideration [7]. The sensitivity of AFB smears obtained from liver tissue has been found to be only 25% [9]. Caseating granulomas are seen in 68% of the cases, whereas highest diagnostic sensitivity of 86% is seen with PCR [9].

The general consensus on treatment is a six-month course of ATT. The initial two months comprise of the intensive phase with isoniazid, rifampicin, pyrazinamide, and ethambutol. For the remaining period, only isoniazid and rifampicin are given [7]. Drug resistance is an emerging problem and it can necessitate the use of more toxic alternative drugs, and the duration of therapy is also extended. Surgery is occasionally indicated for the relief of obstruction and management of fistulae. Intestinal resection, stoma creation and stricturoplasty are the procedures usually resorted to.

## Conclusions

A 22-year-old male presented with a one-month history of fever, night sweats, and weight loss. He was previously healthy, but had history of contact with a TB patient. He had anemia, deranged liver functions, significantly elevated ESR, and a positive Mantoux test. CT of the abdomen revealed multiple microabscesses in the liver and spleen. There was no pulmonary disease. This report represents an atypical manifestation of TB. The patient had isolated hepatic and splenic involvement, and despite the overt clinical and radiologic manifestations, there was no histological or microbiological evidence of infection with TB. Empirical treatment with ATT was started with a successful outcome.

## References

[1] Lienhardt C (2001). From exposure to disease: the role of environmental factors in susceptibility to and development of tuberculosis. Epidemiol Rev.

[2] (2020). Country profiles for 30 high TB burden countries. https://www.who.int/tb/publications/global_report/tb19_Report_country_profiles_15October2019.pdf?ua=1.

[3] Mehta JB, Dutt A, Harvill L, Mathews KM (1991). Epidemiology of extrapulmonary tuberculosis. A comparative analysis with pre-AIDS era. Chest.

[4] Chaudhary P, Bhadana U, Arora MP (2015). Pancreatic tuberculosis. Indian J Surg.

[5] Tanrikulu AC, Aldemir M, Gurkan F, Suner A, Dagli CE, Ece A (2005). Clinical review of tuberculous peritonitis in 39 patients in Diyarbakir, Turkey. J Gastroenterol Hepatol.

[6] Gupta P, Kumar S, Sharma V (2019). Common and uncommon imaging features of abdominal tuberculosis. J Med Imaging Radiat Oncol.

[7] Debi U, Ravisankar V, Prasad KK, Sinha SK, Sharma AK (2014). Abdominal tuberculosis of the gastrointestinal tract: revisited. World J Gastroenterol.

[8] Akhan O, Pringot J (2002). Imaging of abdominal tuberculosis. Eur Radiol.

[9] Hickey AJ, Gounder L, Moosa MY, Drain PK (2015). A systematic review of hepatic tuberculosis with considerations in human immunodeficiency virus co-infection. BMC Infect Dis.

[10] Gupta P, Dhaka N, Rohilla M (2018). Isolated splenic tuberculosis presenting as an unusual splenic mass. Int J Mycobacteriol.

[11] Tang T, Hsu Y, Lee JJ (2014). Disseminated tuberculosis with splenic tuberculosis abscess rupture. A rare presentation. Am J Respir Crit Care Med.

[12] Bhargava DK, Gupta M, Nijhawan S, Dasarathy S, Kushwaha AK (1990). Adenosine deaminase (ADA) in peritoneal tuberculosis: diagnostic value in ascitic fluid and serum. Tubercle.

